# Clinical Depression and Punishment Sensitivity on the BART

**DOI:** 10.3389/fpsyg.2017.00670

**Published:** 2017-05-02

**Authors:** David Hevey, Kevin Thomas, Sofia Laureano-Schelten, Karen Looney, Richard Booth

**Affiliations:** ^1^School of Psychology, Trinity College DublinDublin, Ireland; ^2^Department of Psychology, University of BournemouthBournemouth, UK; ^3^Department of Psychology, St. Patrick’s HospitalDublin, Ireland

**Keywords:** depression, decision making, punishment sensitivity, risk-taking

## Abstract

Depression is associated with altered sensitivity to reward and punishment, which can influence complex decision-making. We examined punishment sensitivity in the performance of participants with major depressive disorder (MDD) with that of a comparison group on the automatic Balloon Analogue Risk Task (BART), which is a direct measure of risk taking. The present study examined the BART performance of 30 individuals with MDD and 30 matched comparison individuals. The comparison group (*M* = 63.25) entered a significantly (*p* < 0.001; *d* = 1.1) higher number of pumps on the BART than the MDD group (*M* = 50.83). Higher levels of depression symptoms were significantly correlated (*r* = -0.40, *p* < 0.05) with entering a lower number of pumps in the MDD group. MDD patients showed an increased sensitivity to punishment on the BART: after a loss, the MDD group decreased (*M* = 13.7) the number of subsequent pumps they entered by a significantly (*p* < 0.001, *d* = 0.81) greater amount than the comparison group (*M* = 4.35). This difference applied to losses only: no difference was found between the groups regarding the magnitude of change in pumps selected after a win. Findings suggest the presence of elevated punishment sensitivity among individuals with MDD, which may contribute to the maintenance of depressive symptoms.

## Introduction

Psychological approaches to depression emphasize the importance of cognitive dysfunction in the disorder, and the Diagnostic and Statistical Manual of Mental Disorders-V (DSM-V; American Psychiatric Association, 2013) considers decision-making impairment a possible symptom for major depressive disorder (MDD). MDD is associated with aberrant reward and punishment learning (e.g., Depue and Iacono, 1989; Must et al., 2006; Steele et al., 2007; Cella et al., 2010; Eshel and Roiser, 2010), and altered sensitivity to reward and punishment may contribute to the maintenance of depressive symptoms (Martin-Soelch, 2009). For example, depressed patients have an enhanced sensitivity to negative feedback and are highly influenced by punishments and losses (Elliott et al., 1996). Punishment sensitivity can be observed during risk-based decision-making tasks, where the individual has to select between multiple options associated with uncertain consequences: when people engage in risk decision making, they pursue some form of reward while exposing themselves to potential punishment (Wallach et al., 1962).

The Balloon Analogue Risk Task (BART; Lejuez et al., 2002) requires repeated decisions for financial gain, and is sensitive to real-world behaviors (Bishara et al., 2009). However, in contrast to tasks such as Iowa Gambling Task (IGT; Bechara et al., 1994) that require participants to learn the reward and punishment contingencies relating to their choic of cards, the BART offers a direct measure of risk-taking. The BART requires participants to make successive pumps within each trial to inflate a balloon. The participant can stop pumping at any time and will win an amount of money depending up on how many pumps of air were put in the balloon: the more pumps you made, the more you could win. However, putting more pumps of air into the balloon increases the risk that the balloon will burst and you will be punished by losing that amount of money. Increased pumping on the BART was significantly related to alcohol and drug use, cigarette smoking, gambling, theft, aggression, and unprotected sexual intercourse in both adolescent (Lejuez et al., 2003; Aklin et al., 2005) and adult samples (Lejuez et al., 2002, 2004). It was also associated with psychopathy and impulsivity among young adults (Hunt et al., 2005). The BART is a reliable (White et al., 2008) simple laboratory task that captures the defining characteristic of risk taking in the real world—when participants pump the balloon, they pursue the reward of monetary gain while exposing themselves to potential punishment with the loss of money.

Of specific interest for the present study, Pleskac et al. (2008) modified the BART wherein the instructions given to participants were altered to inform them explicitly about the best decision strategy to use during the course of the BART (i.e., select 64 pumps) trials to produce the best long-term outcome. However, participants are told that this strategy might not always work and that if 64 is entered, the balloon may still explode before or at that number of pumps. Participants are informed that the balloons may explode at any given pump though they are unaware of the actual explosion point for each balloon. The task was further modified so that instead of making the sequential pumps, the participant now simply entered the number of pumps they wanted to inflate the balloon by and they watched the balloon inflate on screen. If the number the participant enters at the start does not exceed the explosion point, the balloon on the screen inflated by the amount entered and the money they earned was deposited into their “bank account”. However, if the participant typed in a number that exceeds the balloon’s explosion point, the balloon on the screen pops and the participant loses the amount entered.

To date, there has been less research using the automatic BART than the original BART. Research examining the effect of these modifications indicates that they produce unbiased BART statistics (Pleskac et al., 2008). As noted by the developers of the original BART, analysis of only unexploded balloons produced biased results: scores were biased toward low scores, because the more times respondents choose a risky option, the more likely it is that a trial will end in failure. Consequently the authors proposed an adjusted score, which filters out longer response sequences and biases scores toward a lower number of pumps (Pleskac et al., 2008). Furthermore, whereas participants were largely risk averse on the original BART and routinely stop pumping much earlier than is optimal (Lejuez et al., 2002; Wallsten et al., 2005), the modifications have been associated increased risky pump selections by participants (Pleskac et al., 2008). The automatic BART retains similar relationships to substance use indices as the standard version of the task. For example, the automatic BART was associated with increased familial risk of alcohol use (Gorka et al., 2015), and drinking quantity, self-efficacy to control drinking and drinking acceptability (DeMartini et al., 2014). It has also been associated with levels of daily smoking (Larsen et al., 2014), risky driving behaviors and attitudes among adolescents (Vaca et al., 2013).

We compared the performance of participants with MDD with that of a matched comparison group on the automatic BART. We used the automatic BART instead of the original BART as we were primarily interested in how people responded to a loss in the context of being explicitly told the optimal long-term strategy. We were interested to see if participants actually used the optimal strategy consistently (i.e., always enter 64 pumps) or if they used a more risky (i.e., enter higher than 64 pumps) or conservative strategy (i.e., enter lower than 64 pumps) and how their responses changed after reward or punishment. As using the optimal strategy on the BART will occasionally result in losses, we were particularly interested in how the MDD group responded to such losses. In the automatic BART the participants could not avoid losses as there was no way to predict the outcome on individual trials. Based on an assumption of depressed patients’ increased sensitivity to negative feedback, we hypothesized that after a loss, the MDD patients will decrease their next selection significantly more than the comparison group will do after a loss. We did not predict any difference between the MDD and comparison group after a gain.

## Materials and Methods

### Design

A between groups design was used: the BART performance of individuals with MDD was compared to a matched comparison group. The study was conducted in accordance with the Declaration of Helsinki, and was approved by both the School of Psychology, Trinity College Dublin and St. Patrick’s Hospital, Dublin Human Research Ethics Committees. Written informed consent was obtained from all participants after the study’s procedures were fully explained using an information sheet and after the researcher had answered all participants’ questions about the study procedures.

### Participants

Following receipt of ethical approval from the relevant committees, MDD participants were recruited from a clinical service, and comparison participants from the university’s research participant panel. The clinical service is a large urban hospital specializing in mental health problems and participants were adult patients with a current diagnosis of MDD, established by a Structured Clinical Interview for DSM-V (SCID-V; First et al., 2015) administered by a trained psychiatrist. Exclusion criteria, which were determined by the SCID-V and a review of clinical notes, included any preexisting or concurrent co-morbid primary diagnosis that met the DSM-V criteria for bipolar disorders, schizophrenia spectrum and other psychotic disorders, anxiety disorders, obsessive–compulsive disorders, trauma- and stressor-related disorders, and substance-related and addictive disorders. Additional exclusion criteria were acute suicidal or homicidal behavior, personal history of major neurological or physical disorders that could lead to an altered mental state. In addition, all MDD patients scored in the clinical range of the Beck Depression Inventory-II (BDI-II; Beck et al., 1996). All but two of the clinical participants were inpatients. Ten of the participants were patients with first-onset MDD, and others were in the relapse phase. The mean frequency of episodes was 2.67 (SD = 0.85) times. The mean age of onset was 26.43 (SD = 12.59) years old. The mean duration of illness was 86.13 (SD = 70.65) months. In the MDD group, all participants received antidepressant medications (selective serotonin reuptake inhibitors and/or serotonin-norepinephrine reuptake inhibitors).

Once the MDD participants were recruited, purposive sampling was used to recruit from the university’s research participant panel a sample that matched the MDD group. The research participant panel comprises over 1,500 adults from the community who have self-selected to be contacted to participate in research projects. Using the demographic data provided by the panel members, the researchers sought to obtain a sample that matched the MDD group profile in terms of age, gender, and levels of education. Matching was not performed on an individual basis but rather at the group level. The comparison participants were free of any psychiatric or medical condition known to influence cognition and had never taken any form of antidepressant medication, as screened by a self-reporting questionnaire. In addition, comparison patients had to score in the normal range of the BDI-II (Beck et al., 1996) at the testing session.

### Procedure

Comparison participants were tested individually in a laboratory in the university and clinical participants were tested individually in a quiet room in the clinical service. For both groups, an investigator described the study, provided information sheets and consent forms. To motivate participants to perform as well as possible (see Whitlow et al., 2004), in addition to the flat rate of participation of 5 euro, all participants were told that they would get a monetary bonus according to their total earnings on the BART. In fact all participants received the same reward of 10 euro for taking part in the study. The reward was the same as the ethical approval noted that it would be unethical to provide different amounts of money to the clinical population participants based on performance: those who received a lower level of reward compared to others may experience a negative impact on their mood and wellbeing. Given the vulnerable nature of the population we decided to proceed using this approach to prevent any inadvertent distress. Following completion of the BART all participants completed the BDI-II (Beck et al., 1996) to ensure that the groups comprised those scoring in the clinical range (MDD group) and in the normal range (comparison group). Participants were then fully debriefed about the nature of the study and were paid.

### Measures

#### Balloon Analogue Risk Task (Lejuez et al., 2002)

The automatic BART is a computer-simulated assessment of risk taking behavior (Pleskac et al., 2008). A small simulated balloon and balloon pump are presented on a computer screen, and participants have to pump 20 balloons, one at a time. Each pump is worth 1 cent. If they pump too much and the balloon explodes, they lose the money for that balloon. Participants enter the target number of pumps they wish to take at the beginning of the trial (using a yellow dial with digits). Participants are given instructions about the best decision strategy to use, as they are explicitly informed they could win the most money if they pumped 64 times on each trial. The instructions read in part: “*The explosion point varies across balloons, ranging from the first pump to the 128th pump. The ideal number of pumps is 64. What that means is that if you were to make the same number of pumps on every balloon, your best strategy would be to make 64 pumps for every balloon. This would give you the most money over a long period of time. However, the actual number of pumps for any particular balloon will vary, so the best overall strategy may not be the best strategy for any one balloon*”.

They are also provided with feedback after each trial as they are informed of the pump on which the balloon would have exploded on winning trials (i.e., those trials that did not terminate with an explosion) but also on trials ending in explosions. The automatic BART has demonstrated convergent validity with self-reported measures of smoking (Larsen et al., 2014), alcohol (DeMartini et al., 2014), and risky driving behaviors (Vaca et al., 2013).

#### Beck Depression Inventory-II (Beck et al., 1996)

The BDI-II is a 21-item inventory that assesses symptoms of depression over the previous week. The BDI-II has been used extensively in clinical diagnosis and research, and is supported by extensive psychometric literature (Beck et al., 1996). Cronbach’s alpha in the present study was 0.93.

### Data Analysis

*A priori* power calculations using G^∗^Power 3.1.3 indicated that a sample size of 30 per group provided power of 0.80 to detect an ES of 0.67 as significantly different between the groups at 0.05 level (Faul et al., 2007). The ES was hypothesized to reflect a medium to large sized effect. Performance on the BART was measured by the target score, which is the average stated number of pumps for all balloons. This score provides an unbiased estimator of decision-making quality, as the mean for the group should be close to the optimum strategy of selecting 64 pumps. Correlations between variables were performed using Pearson correlations. Comparisons between two groups were performed using independent samples *t*-tests and effect sizes are reported using Cohen’s *d*, with 95% CI. Bayes factors are also reported for non-significant inferential tests. For all analyses, statistical significance was set at *p* < 0.05.

## Results

### Clinical Demographic Profile

The clinical demographic profile of the sample is provided in **Table 1**.

**Table 1:** Demographic profile of the sample.

	MDD group	Comparison group
Gender		
Male	10 (30%)	13 (43%)
Female	20 (70%)	17 (57%)
Highest education level completed		
Primary	10 (33%)	8 (27%)
Secondary	17 (57%)	18 (60%)
Higher level	3 (10%)	4 (13%)

There were no significant differences between the groups in relation to gender [χ^2^ (1, *N* = 60) = 0.64, *p* = 0.28; Bayes factor = 1.23] or education level [χ^2^ (2, *N* = 60) = 0.39, *p* = 0.82; Bayes factor = 1.21]. Furthermore, there was no significant difference in the age of the MDD group (*M* = 39.47; *SD* = 12.20) and the comparison group (*M* = 38.13; *SD* = 11.35), *t*(58) = 0.66, *p* = 0.66, Cohen’s *d* = 0.11; 95% CI = -0.39 to 0.62; Bayes factor = 1.87.

### Relationship between the BART and BDI-II

Examination of the scatter plot between the BART and BDI-II scores among the MDD group showed that their relationship could be modeled as linear: a Pearson correlation test showed that the BART target score significantly correlated with the BDI-II [*r* (28 *df*) = -0.40, *p* < 0.05]. The negative correlation indicated that higher levels of depression symptoms were associated with entering a lower number of pumps (i.e., lower BART target score).

### BART Performance

The MDD group (*M* = 50.83, *SD* = 14.62) made entered significantly lower number of pumps on the BART than the comparison group (*M* = 63.25, *SD* = 11.29), t(58) = 3.68, *p* < 0.001; Cohen’s d = 0.95; 95% *CI* = 0.41–1.47. The BART target score mean of the comparison group was close on average to the optimum value of 64: a one-sample *t*-test showed no significant difference between the group mean and the optimum value of 64, *t*(29) = -0.67, *p* = 0.51; Bayes factor = 1.10. However, the MDD group average response was significantly lower than 64, *t*(29) = -4.17, *p* < 0.001. There was no significant difference between the groups in relation to the rate of balloons saved: the MDD saved 57% (*SD* = 7.97) whereas the comparison group saved 53% (*SD* = 10.36), *t*(58) = 1.70, *p* = 0.09; Bayes factor = 1.36.

**Table 2** illustrates the pattern of responding over the 20 trials split into quartiles. In the initial trials both groups varied considerably around the optimal value of 64. By the end of the task the comparison group were on average entering pump values close to the optimal value (last five pumps *M* = 65.36), whereas the MDD group on average continued to select sub-optimal values (last five pumps *M* = 52.87).

**Table 2:** M (SD) automatic BART pumps per quartile by group.

Group	Pumps 1–5	Pumps 6–10	Pumps 11–15	Pumps 16–20	Pumps overall
MDD	59.37 (14.32)	43.26 (15.74)	47.82 (14.39)	52.87 (13.88)	50.83 (14.62)
Comparison	65.41 (12.36)	60.14 (11.31)	62.13 (12.68)	65.36 (10.22)	63.25 (11.29)

### Punishment Sensitivity

**FIGURE 1:**
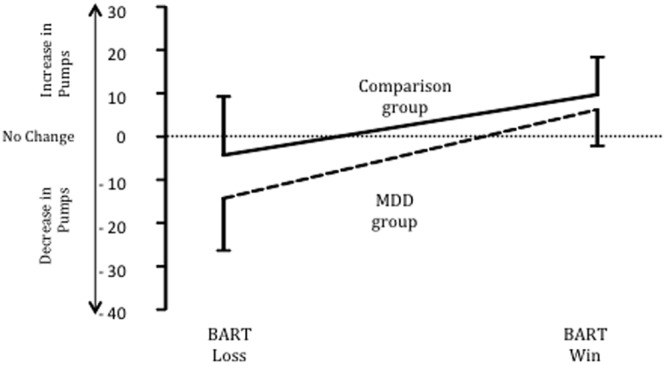
**Changes (M ± SD) in BART pumps after a loss and a win for MDD and comparison groups**.

Punishment sensitivity was examined by comparing the groups on the average magnitude of change in pumps selected after a loss on the BART (see **Figure 1**). The MDD group decreased the number of pumps (*M* = 13.7; *SD* = 9.12) they entered after a loss by a significantly greater amount than the comparison group (*M* = 4.35; *SD* = 11.5), *t*(58) = 3.49, *p* < 0.001, Cohen’s *d* = 0.90; 95% CI = 3.99–14.71. This difference applied to loss only: no difference was found between the MDD (*M* = 6.01; *SD* = 7.87) and comparison group (*M* = 9.65; *SD* = 8.55) regarding the magnitude of change in pumps entered after a win, *t*(58) = 1.71, *p* = 0.11; Cohen’s *d* = 0.44, 95% CI = -0.95 to 0.07. In addition, after a loss, the 83% of MDD decreased their value on the next selection whereas only 44% of the comparison group decreased their value on the subsequent selection, χ^2^(1, *N* = 60) = 10.34, *p* < 0.001.

## Discussion

The MDD group showed an increased sensitivity to punishment on the BART: after a trial where they were punished by losing money, the next number of balloon pumps they entered was much lower than their previous number. Whilst such a strategy may reduce the risk of subsequent punishment, it also entails a loss of potential reward. Even when presented with the optimal value to enter, the MDD group on average was significantly below this value. The negative correlation between the BDI and the BART target score indicates that those participants with more depression symptoms entered lower pump values on the BART, which may reflect a strategy to avoid the possible punishment associated with higher values. Of note, a study examining the effects of alcohol on performance on the automated BART revealed that in comparison to those drinking placebo, the alcohol group did not approach the optimal level of 64 pumps, but kept on using a less effective strategy of entering lower pumps per balloon (Euser et al., 2011)—this mirrors the pattern observed in the present study by the MDD group.

The pattern of pump value entries by the MDD group is consistent with a punishment avoidant strategy. Consequently, this impairs their ability to focus on the rewards and to develop adaptive rewarding strategies. It has been noted that positive events such as winning money may fail to adequately reinforce behavior in people with MDD (Henriques and Davidson, 2000), as depressed patients fail to perceive rewards as reinforcing due to low hedonic capacity (Meehl, 1975). MDD has been characterized by significant changes in both motivational and affective processing (Leppänen, 2006). Dominated by persistent dysphoric emotions and thoughts (anhedonia) such patients can exhibit a decreased motivation to seek and a reduced ability to experience reward (Drevets, 2001). Those with depression may be sensitive to the saliency of punishing stimuli, and they may act to minimize exposure to such punishing outcomes.

Although avoiding punishments may be adaptive in some circumstances, the continual use of a strategy in a rigid or context-insensitive manner is likely to reduce the probability of being exposed to rewarding environments, which, in turn, may exacerbate depressive symptoms (Smoski et al., 2008). Thus, avoiding risk often will lead to missed opportunities for rewards. Engaging in an increasing number of rewarding experiences has been shown to be beneficial for improving depression; furthermore, behavioral activation as part of cognitive-behavioral therapy requires people to increase behaviors that lead to a sense of mastery and self-efficacy, as well as those that result in pleasurable consequences (Jacobson et al., 1996). However, behavioral activation involves the potential for reward as well as punishment. For example, going out and getting involved in social activities carries the possibility of both positive (e.g., making friends, strengthening relationships, engaging in enjoyable activities) and negative consequences (e.g., rejection, social disapproval, criticism). Depressed individuals may be particularly attuned to and avoidant of the negative consequences of these activities, and consequently, may choose not to take the risk. Thus, if generalized, this punishment avoidance strategy may be an important factor in the maintenance of depression (Chapman et al., 2007). Punishment sensitivity has been associated with depression levels (Eshel and Roiser, 2010); it has been argued that avoiding decisions that have potential negative consequences reflects an underlying process impacting on depression and psychological flexibility (Leahy et al., 2012). Additional research examining such processes is warranted.

The neurobiological basis of punishment sensitivity remains to be determined (Kim et al., 2015): punishment-based learning is associated with greater activation in the insula and lateral orbitofrontal cortex (Wächter et al., 2009; Elliott et al., 2010; Xue et al., 2013). Furthermore, dopamine plays a role in the formation of behavioral learning strategies aimed at avoiding aversive stimuli (Ilango et al., 2012).

The MDD group self-selected to participate in the study, were predominantly inpatients, and were on antidepressant medications, which can affect sensitivity to reward and punishment; antidepressants may enhance sensitivity to negative outcomes so that they can be perceived and avoided (Dalgleish et al., 2004). Consequently the effect of the medication on performance is unclear as we did not record the dosage at the time of the study. The strength of drug may impact on performance and future research should examine the nature of this relationship. In addition, the study did not use self-report psychometric instruments to assess loss aversion, impulsive personality traits and risk tendencies in the two groups; this limits the capacity to rule out the possible confounding effects of these traits on BART performance. Given the relationship between impulsivity and depression among clinical populations (Jakubczyk et al., 2012; Dervic et al., 2014) future research should include such measures.

In line with previous decision making studies (e.g., Whitlow et al., 2004), a performance-related monetary reward was offered to enhance participant motivation to perform successfully; however, this may not have proved sufficient to engage the participants, as depressed patients are characterized by lack of appropriate responses to rewards (e.g., Layne, 1980). Although the comparison group all scored in the normal range on the BDI-II and did not self-report any history of psychiatric and medical conditions that could impact on performance, a psychiatric diagnostic interview (e.g., SCID-V) was not used to confirm their mental health status. In addition, the comparison group were a self-selected group of community dwelling adults who were interested in taking part in psychological research.

## Conclusion

Individuals with MDD engaged in punishment-sensitive decision-making on the automatic BART: although this strategy can minimize potential negative outcomes it comes at the cost of minimizing potential rewarding outcomes, which may contribute to the maintenance of depression.

## Author Contributions

DH wrote the paper, analyzed the data, and contributed to the design of the study. KT, KL, and RB contributed to the paper and the design of the study. SL-S contributed to the paper, collected the data, and the design of the study.

## Conflict of Interest Statement

The authors declare that the research was conducted in the absence of any commercial or financial relationships that could be construed as a potential conflict of interest.
